# Oral Manifestations of Crohn’s Disease: A Systematic Review

**DOI:** 10.3390/jcm12206450

**Published:** 2023-10-10

**Authors:** María Pilar Pecci-Lloret, Emma Ramirez-Santisteban, Adraz Hergueta-Castillo, Julia Guerrero-Gironés, Ricardo Elías Oñate-Sánchez

**Affiliations:** Gerodontology and Special Care Dentistry Unit, Morales Meseguer Hospital, Faculty of Medicine, IMIB-Arrixaca, University of Murcia, 30008 Murcia, Spain; mariapilar.pecci@um.es (M.P.P.-L.); emma.ramirezs@um.es (E.R.-S.); adraz.hergueta@um.es (A.H.-C.); reosan@um.es (R.E.O.-S.)

**Keywords:** Crohn’s disease, oral manifestations, oral lesions, systematic review

## Abstract

Crohn’s disease (CD) is a chronic inflammatory intestinal condition that can affect the entire gastrointestinal tract. It is characterized by its clinical heterogeneity and irregularities in its course. The etiology and pathogenesis are not well established, so it is difficult to establish an early diagnosis and an effective treatment plan. The objective of this systematic review was to present a qualitative synthesis of the studies referring to the oral manifestations of CD. This systematic review was carried out following the PRISMA guide. Research was conducted in the Pubmed, Web of Science, Scopus, Scielo, and Cocrahne Library databases on 23 February 2023, and updated on 1 September 2023. Articles published between 2012 and 2023 were selected. Articles that analyzed the oral manifestation of CD patients and met the established search terms. In addition, the quality of all the selected studies was analyzed following the CARE guidelines for case reports and the STROBE scale for observational studies. A total of 19 articles were included in this review that met the inclusion criteria. Regarding the oral manifestation of CD, oral ulcers, angular cheilitis, and gingivitis stand out. Periodontitis and vegetative pyostomatitis were the least representative manifestations. The most prevalent locations were lips, mucosa, and gingivae. Ulcers, gingivitis, and angular cheilitis are the most frequent oral manifestations in patients with CD. Their early identification and possible relationship with the disease are important for an early diagnosis and an adequate treatment plan.

## 1. Introduction

Crohn’s disease (CD) is described as a chronic inflammatory bowel disease, mainly affecting the lower gastrointestinal tract, with the ileocolonic region being the most affected. It is characterized by periods of remission and exacerbation. It is incurable and has a disabling course due to the development of various complications. In addition, it involves the skin, the musculoskeletal system, and the eyes [1,2,3,4].

In terms of epidemiology, CD affects men and women equally. Its age of onset has a bimodal distribution, with a peak between 20 and 40 years of age and a second peak between 50 and 60 years of age. The disease’s incidence and prevalence have increased significantly worldwide [3].

Crohn’s disease is of unknown etiology, is associated with an altered immune response, and has a strong genetic association. Whole genome studies revealed that when the NOD-2 receptor (a microbial recognition protein expressed on monocytes, macrophages, dendritic cells, and paneth cells) is mutated, an alteration of the patient’s immune response occurs and is directed towards the bacterial flora of the gut, being related to the development of CD [2,5,6,7].

In addition to the genetic susceptibility of patients, the interaction with environmental factors has to be present. CD appears to be the result of an alteration in the commensal microbiota of the gut. This may be altered by diet, drug use, smoking, or infectious processes. The most relevant would be Mycobacterium avium paratuberculosis (MAP), or measles virus. In addition, recent studies link the use of toothpaste or a history of appendectomy as environmental factors related to the disease. On the other hand, it has been shown that breastfeeding or contact with animals in childhood may be protective factors against the disease. However, there is no causal relationship between the above-mentioned factors and the development of the disease [2,3,5,6].

The presentation of the disease is very heterogeneous, with many subtypes. Initially, the most frequent presentation is purely inflammatory. In the final presentation, intestinal fibrosis is found, leading to intestinal strictures and fistulas. There is a 50% chance of developing perianal fistulas within 20 years of the initial diagnosis [8,9].

The main symptoms that raise suspicion of Crohn’s disease are abdominal pain and chronic diarrhoea leading to weight loss, observed in 60–70% of patients. Depending on the type of diarrhoea emitted by the patient, the area that is most affected may be suspected. If the diarrhoea is large in volume, the involvement is more likely to be located in the ileum, whereas if the diarrhoea is smaller and mucus and blood are present, the colon is possibly the area most affected [2,6,9].

Oral manifestations are common in Crohn’s disease and may present as the first symptom. Early symptoms include aphthous ulcers, redness, edema, and pain. They are predominantly located on the mucosa, lips, and tongue. The presence of lesions in the cavity was associated with an exacerbation of the disease. There are specific and non-specific manifestations. The difference lies in the non-caseating inflammation and granulomatous substrate in the case of specific manifestations. Specific manifestations include ulcers and lips with granulomatous changes or Miescher cheilitis. Sometimes indurated polypoid tumours can be observed on the buccal mucosa in their specific form. Non-specific manifestations include oral aphthous ulcers, erythema nodosum, and a variety of neutrophilic dermatoses [1,2,10].

Treatment of CD is primarily aimed at achieving sustained clinical and endoscopic remission. It is important to interrupt the destructive course of the disease, which, if prolonged, can lead to intestinal failure and associated complications. It should be individualised for each patient [5].

Finally, it is important to emphasise the importance of early and accurate diagnosis and subsequent appropriate treatment. There is a high risk of malignancy for people with Crohn’s disease for small bowel, colorectal, and mucinous carcinoma arising from perianal fistulas [9].

Currently, CD is a chronic intestinal disease of unknown aetiology, but with patients with high genetic susceptibility and involvement of various environmental factors, some of which have no proven scientific evidence. There is variability in its clinical presentation, ranging from intestinal and extraintestinal manifestations to associated autoimmune disorders. This makes a diagnosis and choice of treatment difficult.

There are several oral clinical manifestations that appear as early signs of the disease and that are essential to detect in order to identify the disease as early as possible. This leads to an improvement in the patient’s prognosis and quality of life. Therefore, there is a need for a multidisciplinary team for its management that is aware of the most characteristic manifestations, and in this case, at the oral level. There is a lack of scientific knowledge on the subject to date. Thus, the aim of this systematic review was to present a qualitative synthesis of various studies concerning the oral manifestations of Crohn’s disease.

## 2. Materials and Methods

### 2.1. Declaration and Protocol

This systematic review was conducted according to the PRISMA guide, an acronym for “Preferred Reporting Items for Systematic Reviews and Meta-Analyses”. In addition to the regulations of the Final Degree Projects of the University of Murcia. It was registered in PROSPERO with registration number CRD42022377915.

### 2.2. Inclusion and Exclusion Criteria

The criteria for the inclusion of articles were the following: (i) articles published between the last 10 years (2012 and 2023); (ii) articles analysing and identifying oral manifestations present in CD patients; (iii) articles published in English or Spanish; (iv) any type of research article.

Exclusion criteria were as follows: (i) articles published more than 10 years ago; (ii) articles that provided information about inflammatory bowel diseases in a generalised way and did not study the oral manifestations of CD; (iii) articles in a language other than English or Spanish; (iv) systematic reviews or literature reviews.

In order to establish the inclusion criteria, the PICO model should be followed:

Population/problem (P): Patients with Crohn’s disease; Intervention (I); Comparison/control (C): Healthy patients; Outcome (O): Oral manifestations present in patients with Crohn’s disease.

So, the PICO question is: What are the oral manifestations of patients with Crohn’s disease?

### 2.3. Search Strategy

#### 2.3.1. Sources of Information

In order to search for information on the topic proposed for this systematic review, an exhaustive search was carried out in the following databases: Pubmed, Web of Science, Scopus, Scielo, and Cocrahne Library.

This search was carried out on 23 February 2023 and updated on 1 September 2023.

#### 2.3.2. Search Terms

The terms used for the search were obtained from the Mesh (Medical Subject Heading) thesaurus. Those referring to Crohn’s disease are “Crohn’s disease”, “Crohn’s”, “Crohn’s”, “inflammatory bowel disease”, and “bowel disease.” Those referring to oral manifestations are “Oral manifestation” and “oral lesion”. Boolean operators (“AND” and “OR”) were used to relate the mentioned terms to each other. The following table shows the results obtained from the search performed (Table 1).

#### 2.3.3. Selection of Studies

The studies obtained in the search process were entered into the Endnote Analytics bibliographic manager. After that, the manager discarded duplicate articles, and other duplicate articles not identified by the manager were manually discarded.

Subsequently, a first selection of articles was made on the basis of their titles. The abstract of the articles selected by title was read, and a second selection was made. Finally, the selected articles were read in full text and checked for compliance with the inclusion and exclusion criteria.

#### 2.3.4. Data Extraction

For the bibliometric analysis, the years of publication, authors, city, and journals were taken into account. To summarise the methodology of the studies, a summary table was drawn up with the following data: type of studies; most frequent manifestations; most frequent location in the oral cavity.

#### 2.3.5. Quality Analysis

The quality of the studies included in this systematic review was analyzed by two reviewers (ERS and MPPL). Any discrepancies were decided by involving a third reviewer (JGG). Use was made of the CARE guideline for clinical cases, which sets out a series of recommendations on the quality of clinical cases. It is composed of 30 items, 11 of which refer to the different parts that make up a clinical case (title, keywords, abstract, introduction, patient information, clinical findings, dates, diagnostic evaluation, therapeutic intervention, follow-up and results, discussion, patient perspective, and informed consent). Each of these was marked with a positive tick (✔) when the requirement was met and a cross (✘) as negative when the requirement was not met. The attached table shows the 30 items contained in the CARE guide that were followed for our analysis. In the case of control, cross-sectional, cohort, and prospective studies, the guide used was the modified STROBE scale (Strengthening the Reporting of Observational Studies in Epidemiology), which establishes the necessary recommendations for what an observational study should include. It is composed of 11 items that refer to the parts of the studies mentioned (material, methods, and results). Studies with 9 to 11 items were selected as low bias; those with 6–8 were considered moderate bias; those with less than 5 were considered high bias. The same methodology as above was applied.

## 3. Results

### 3.1. Selection of Studies and Flow Diagram

**Figure 1:**
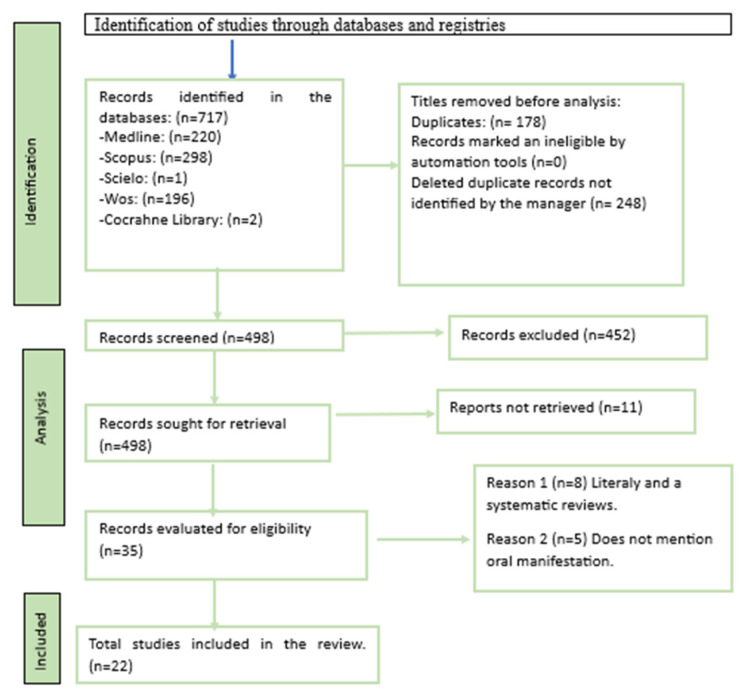
Flowchart diagram.

The results of the selection are shown in Figure 1. After the exhaustive database search, a total of 717 references were obtained, of which 220 were from Medline, 298 from Scopus, 196 from Web of Science, 2 from Cocrahne Library, and 1 from Scielo.

Later, 178 articles were discarded using the Endnote bibliographic manager. Subsequently, another 248 duplicate references not detected by the manager were eliminated, resulting in a total of 498 articles. Next, 452 articles that did not cover the topic of study were eliminated by title and abstract. Thus, 46 references were evaluated in full text, discarding 24 of them and finally obtaining 22 valid articles.

### 3.2. Bibliometric Analysis

The studies resulting from the search were distributed by year of publication, country of publication, and journal of publication. With regard to the year of publication, the continuous publication of articles on the subject is perceived, with an increase in the year 2021. The years 2019 and 2020 are the years with the lowest publication rate. Concerning the country of publication, the highest prevalence of published studies is found in the United States with seven articles, followed by Italy with four published articles. For the rest of the countries mentioned, one publication per country was found. There are a wide variety of journals that include articles related to CD and its oral manifestations, the most prominent being BMJ, which, in addition to case reports, also published a case control, and Wiley Clinical, which includes 2 case reports.

### 3.3. Results of Data Extraction

The results of the extraction are represented in Table 2, where the different categories mentioned above and the significance of the association of oral manifestations as a clinical part of CD can be observed.

The results obtained are shown in Table 2. As can be seen, most of them are clinical cases, with a single patient in all of them. There are fewer observational studies, but they contain a larger volume of patients, with a maximum of 113 patients [13].

The age of the patients in the studies varied. Patients ranged in age from 6 to 64 years of age [10,14]. It should be noted that most of them were in their early twenties [15,19,20,28].

All of them had oral lesions. The evolution of these lesions is unknown in some cases, but in the cases in which it was known, the minimum duration was 1 month [21,23]. The clinical case with the longest evolution of the lesions was 30 years old [14].

The most prevalent oral manifestations were angular cheilitis, oral ulcers, and gingivitis, as well as pain, dysphagia, halitosis, bleeding, and inflammation. Periodontitis and vegetating pyostomatitis were the least representative manifestations.

Regarding their location, the lips, mucosa, and gingiva are the places where most of them were found. However, some patients are shown with lesions at the level of the floor of the mouth, posterior mandible, palate, or even the uvula. Some of the cases presented were diagnosed with CD at the time of the oral manifestations, while others were diagnosed with CD as a result of the oral manifestations. The described lesions were examined microscopically, and the findings were summarised as diffuse non-caseating granulomatous inflammation with lymphocytic infiltrate and the presence of multinucleated giant cells, monocytes, and plasma cells.

In several patients, a differential diagnosis of the oral manifestations presented was necessary. The most repeated diseases were orofacial granulomatosis, sarcoidosis, and pemphigus. Tuberculosis, syphilis, vasculitis, or infectious diseases were mentioned as possible diagnoses before determining the final diagnosis of CD.

Finally, the medication administered to patients was mainly directed at CD and not at the oral manifestations. In the same way that it improved the course of the disease, it reduced the symptoms at the oral level, and in many cases, the manifestations found disappeared. We highlight the administration of immunosuppressants (azathioprine) and monoclonal antibodies (infliximab and adalimumab) as first-line treatments.

### 3.4. Quality Assessment

The quality of the clinical cases was assessed using the CARE guideline [32] (Table 3). The quality of the studies was medium, as many of them did not meet items 8b, 9c, 10c, 10d, and 12 referring to therapeutic intervention, follow-up, outcomes, and patient perspective. Only items 6–8 referring to timing, clinical findings, and diagnostic assessment were fulfilled by all items [10,12,14,17,23,25,28,30,31].

The articles were evaluated, after which a percentage was established for each of them, with the highest percentage achieved being 83.33%, followed by 76.66% [16,26]. The clinical cases with a higher bias have a lower percentage, and in this case, their value is 23.33% [12]

In the case of observational studies, the modified STROBE scale was used (Table 4). The quality of the studies was moderate with a high bias [11,13,24,29]. Item 5 was not met by any of the studies, while items 1 and 7 were met by all of them (Table 4).

## 4. Discussion

The results of this systematic review indicate that all selected studies showed oral manifestations of CD.

Oral manifestations are part of the extraintestinal manifestations of CD. It is vitally important to know about them and the prevalence in which they occur. In the adult population, lesions are found in around 0–9%, while in children, they appear in around 50–80% of patients and may precede severe intestinal involvement in up to 42% of the latter [23,28,30,31]. Rarely have cases been reported where oral involvement was unique and unaccompanied by intestinal disease [14].

The oral cavity is recognised as a useful part of the gastrointestinal tract in the diagnostic procedure for CD. Thus, dentists play an important role in the early diagnosis of the disease and subsequent improvement in both quality of life and prognosis, especially in the paediatric population, with a major impact on growth, puberty, and emotional development [25,33].

Oral involvement can take two forms: specific, including diffuse lip and oral swelling, cobblestoning of the oral mucosa, linear and serpiginous ulcers, and mucogingivitis. In the paediatric population, these types of lesions are more common, with mucogingivitis and ulcers being predominant, and they are non-specific, differentiated from the previous ones by the lack of granulomas when observed microscopically. These include aphthous ulcers, angular cheilitis, and glossitis. It should be noted that these ulcers are sometimes not part of the disease process but are secondary to nutritional deficiencies caused by the course of the disease [10,11,15,17,20,24,27,28,31].

Periodontitis and vegetating pyostomatitis are non-specific manifestations that only some articles classify as such [23,27,30]. CD is one of the systemic conditions affecting the periodontium. Recent studies have speculated that CD is likely to induce periodontitis by changing its oral microbiota and subsequent inflammatory response [27,29].

A 2012 study by Szczeklik et al. [11] found a higher prevalence of periodontitis in patients with CD. One year later, a case-control study conducted by Dr. Stephan Vavircka et al. [13] affirms the previous findings and provides that, in addition to the higher prevalence, the disease behaves with greater severity and extension, both in the adult population. Recently, the first case of a girl with CD presenting with periodontal disease and advanced bone loss has been described [11,27].

In addition, it is important to recognise vegetating piostomatitis. A very specific marker of CD, it is a very rare oral disorder characterised by white or yellow pustules affecting the oral cavity on an erythematous base, which, when ruptured, take the form of a snail’s footprint [14,21].

Regarding the location of the lesions mentioned, there is a predominance of lesions on the mucosa, especially on the lower lips and gums. Lesions are present in other less common locations, such as the soft palate, uvula, tongue, and pillars of the fauces [10,11,12,13,14,15,16,17,19,20,21,22,23,24,25,26,27,28,30,31].

Finally, it can be concluded that the most predominant oral manifestations of CD are mucosal cobblestoning, the presence of linear ulcers, granulomatous cheilitis of the lips, with a predominance of the lower lip, and mucogingivitis. However, some less conspicuous but indicative lesions are found, such as vegetating pyostomatitis or periodontitis in some cases.

As well described, mucosal cobblestoning is significant in CD. Macroscopically in the intestine, one can observe the onset of small focal ulcers that coalesce to form longitudinal, serpiginous ulcers, giving the cobblestone appearance mentioned at the oral level. Microscopically, we find chronic patchy and transmural inflammation, with an increase in plasma cells and lymphocytes, discontinuous irregularity of the crypts, but without rupture of the crypts, and the presence of non-caseating granulomas in the lamina propria. In oral biopsies of the reported cases, plasma cell and lymphocyte infiltrate and the presence of non-caseating granulomas of the lamina propria and submucosa were found in most of them [6,10,12,14,15,16,17,19,20,22,23,25,28,30,34].

It is important to note that the presence of non-caseating granulomas is specific and may fit various diagnoses such as orofacial granulomatosis, although according to Colin E. McCorkle et al. [31], author of one of the selected studies, orofacial granulomatosis is a sign of an underlying process and not a definitive diagnosis as described by other authors [10,14,17,19,24,30]. Therefore, it could be suggested that oral ulcers share histology and appearance with intestinal ulcers.

The presenting symptomatology is varied. In many of the articles mentioned, the patient presented with pain, mostly caused by ulcers. Others presented with lip enlargement, gingival irritation, dry lips, and cracking. The proportion of asymptomatic patients was high, which complicates the early diagnosis of the disease, as the user attended the doctor when the lesion was very evident. Sometimes, oral symptomatology was accompanied by intestinal symptoms such as diarrhoea, weight loss, abdominal pain, and general malaise [10,12,14,15,17,18,19,20,21,23,24,26,27,28,31]. There are several treatments for CD, but the most widespread is pharmacological. We highlight the use of aminosalicylates, corticosteroids, immunomodulators, and biological therapy [10,11,12,13,14,15,16,19,20,21,22,23,24,25,26,27,28,30,31]. Most patients with oral lesions were treated with systemic therapy; only three cases were reported where lesions were treated with topical corticosteroids. Two of them received only topical corticosteroid rinses, showing marked improvement in symptomatology and eventually remission. The author of the third case, Victoria Woo et al. [10], notes that the patient received azathioprine and aminosalicylates in addition to topical treatment, which also improved intestinal symptoms. Saede Atarbashi-Moghadam et al. [21] stress the importance of using local treatment in the absence of systemic symptomatology but note that it has limited success [14,17,21,26]. In several articles mentioned, patients were treated with immunosuppressants and corticosteroids [10,11,12,13,14,16,19,20,22,23,24,25,26,27,28,31]. The results were very good, as there was an improvement in oral and gastrointestinal lesions. Long-term use of immunosuppressants can present severe complications for the patient. Harim Tavares dos Santos et al. [22] report a rare case of paracoccidioidomycosis in a woman with CD on long-term azathioprine treatment who presented with a blackberry lesion on the palate. The use of immunosuppressants leads to a series of complications, including fungal infection, virus, leukopenia, or worsening of the periodontal status and overgrowth in the case of mercaptopurine, according to Mei Li huang et al. [12,20,25,27,30].

Finally, biologic therapy is one of the most effective and accepted therapies for CD. The most widely used are Adalimumab, infliximab, and ustekinumab [11,13,15,16,24,26,27,28,31]. Patients treated with Adalimumab, in the articles selected for this study, have been the group with the most complications and recurrence of symptoms [16,28,31].

Preidl et al. [16] report a case of osteonecrosis of the jaw in a patient with CD after a course of bisphosphonate therapy and current treatment with Adalimumab. In other case reports, some correlation between patients with osteonecrosis of the jaw and biologic therapy can be seen [35]. It is still unclear whether biologic therapy interferes with bone physiology, bone turnover, and long-term wound repair.

This review has limitations, as do the other published articles. Limitations in the selection of the studies included in the systematic review have to be considered. Most of the articles selected were case reports of medium quality. With respect to the observational studies chosen, they were smaller in number and highly biased. Thus, the articles are biased, which is a drawback for the presentation of good-quality articles.

The main limitation of this review is the number of patients. Most of the proposed articles are clinical case reports of a single patient. In the case of observational studies, the number of patients reaches a maximum of 113, but only 69 of them had CD. Even in one of them, the oral examination was not performed by dentists [13]. Moreover, in most of them, the follow-up of the patient after the healing of the lesions was not described.

As mentioned earlier in this review, knowledge of the oral manifestations of patients with CD and collaboration between gastroenterologists and dentists will help in the early diagnosis of the disease and subsequent improvement in both the prognosis and quality of life of patients, as has already been seen with other diseases and their oral manifestations [36].

## 5. Conclusions

CD is a chronic, relapsing inflammatory bowel disease with uncertain pathogenesis. The aetiology is speculated to be multifactorial and involves genetic, environmental, immune, and microbial factors. It affects the entire gastrointestinal tract, but lesions predominate in the terminal ileum and colon. It is relapsing, and there is currently no cure for it.

Based on the results obtained in this systematic review, we can conclude that:

- CD presents with oral manifestations. Some oral lesions develop silently and go unnoticed by the patient. Others become established, causing pain and incapacitating the patient in their normal life, sometimes accompanied by gastrointestinal symptoms.
- Oral involvement in CD patients has been reported in 0.5% to 37% of cases. The length of time in the mouth is not well defined, as in some patients the lesions go unnoticed and in others there are recurrences.
- The most representative oral manifestations of CD are mucogingivitis (especially in the paediatric population), angular cheilitis, serpiginous and linear ulcers, and cobblestone-like mucous membranes.
- Visualisation of oral manifestations of CD plays an important role in the early diagnosis of CD, as they are sometimes the first sign of the disease or oral and intestinal lesions are found simultaneously.

More studies with better quality are needed to corroborate the results obtained in this work.

## Figures and Tables

**Table 1:** Search fields.

Database	Search Field	Results
Medline (PubMed)	1# “Crohn’s disease” OR “Chron’s” OR “Crohn” OR “inflammatory bowel disease” OR “bowel disease”.	103,567
	2# “oral manifestation” OR “oral lesion”.	9302
	1# AND 2#	220
Web of Science	1# “Crohn’s disease” OR “Chron’s” OR “Crohn” OR “inflammatory bowel disease” OR “bowel disease”.	136,376
	2# “oral manifestation” OR “oral lesion”.	6826
	1# AND 2#	196
SCOPUS	1# “Crohn’s disease” OR “Chron’s” OR “Crohn” OR “inflammatory bowel disease” OR “bowel disease”.	153,087
	2# “oral manifestation” OR “oral lesion”.	11,159
	1# AND 2#	298
Scielo	1# “Crohn’s disease” OR “Chron’s” OR “Crohn” OR “inflammatory bowel disease” OR “bowel disease”.	617
	2# “oral manifestation” OR “oral lesion”.	1065
	1# AND 2#	1
Cochrane Library	1# “Crohn’s disease” OR “Chron’s” OR “Crohn” OR “inflammatory bowel disease” OR “bowel disease”.	8479
	2# “oral manifestation” OR “oral lesion”.	278
	1# AND 2#	2

**Table 2:** Description of the variables differentiated for each of the articles examined.

Author	Study Type	Participants Number	Age	Oral Manifestations	Location	Treatment	Developments	Anatomical Diagnosis	Differential Dx
Katarzyna Szczeklik et al., 2012 [11]	Prospective Study	95	-------	Glossitis, ulcers, canker sores, aphthae, gingivitis and angular cheilitis	Tongue, gingiva and mucosa	Mesalazine, azathioprine, corticosteroids, tumour necrosis factor	-----------	---------------------	----------------------
Nicolas Bruscino et al., 2012 [12]	Clinical Case	1	12 y	Perioral erythema, vesicles, pustules, crusts and swelling	Lower lip	Mercaptopu- rina	4 years	Non-caseating granulomas, lymphocytes and plasma cell infiltrate	----------------
Stephan R. Vavricka et al., 2013 [13]	Case Control	113	40 y	Gingivitis, periodontitis, ulcers, leukoplakia and angular cheilitis	Lips, gums, palate and tongue	Thiopurines Anti-TNF therapy	----------	--------------------------	-----------------
Hamid Salek et al., 2013 [14]	Clinical Case	1	64 y	Painful cobblestone ulcers	Oral and labial mucosa	Topical corticosteroids	30 years	Granulomatous inflammation	Orofacial granulomatosis, vegetating pyostomatitis, chronic non-specific ulcers, fungal infection, tuberculosis and pemphigus.
Carolina ciacci et al., 2014 [15]	Clinical Case	1	23 y	Granulomatous cheilitis	Lowerlip	Infliximad	-----------	Inflammatory tissue with non-caseating granulomatous inflammation	----------------
Raimund HM Preidl et al., 2014 [16]	Clinical Case	1	36 y	Perimandibular swelling, Dysphagia, and pain	Mandibular posterior region	Prednisolone Adalimumab	-----------	Granulomatous inflammatory reaction with necrosis	-------------
Bn Padmavathi et al., 2014 [17]	Clinical Case	1	34 y	Angular cheilitis, Swelling and hyperplasia	Lips and buccal mucosa	--------------------	13 years	Presence of diffuse lymphocytes and clusters with scattered fibrotic aggregates of non-caseating granuloma	Sarcoidosis Orofacial granulomatosis
G gale et al., 2015 [18]	Cohort study	29	32 and 7 y	Swelling, erythema, enlargement gingival and pain	Lips, mucosa and gingiva	--------------------	------------	Non-caseating granulomas with multinucleated giant cells, lymphoedema and lymphocytic infiltration	Orofacial granulomatosis
Victoria L. Woo, 2015 [10]	Clinical Case	1	6 y	Pain, bleeding, erythema and ulcers	Gum	Mesalamine Azathioprine	7 months	Fibrous connective tissue with lymphocytes and plasma cells+ non-caseating granulomas	Sarcoidosis Orofacial Granulomatosis
Henedina Antunes et al., 2015 [19]	Clinical Case	1	17 y	Painful ulcers and cheilitis	------------	Prednisolone Azathioprine	2 months	Lymphoplasmacytic infiltrate and epitheloid granulomas	Orofacial Granulomatosis
Bryce L. Desmond et al., 2016 [20]	Clinical Case	1	17 y	Granulomatous cheilitis of the inf. lip	Lip inf.	Azathioprine	---------	Granulomatous dermatitis with lymphocytes + plasma cells	Idiopathic granulomatous cheilitis
Saede atarbashi-moghadam et al., 2016 [21]	Clinical Case	1	39 y	Pain, halitosis ulcers and exophytic pustules	Gingiva and mucosa	Mezalanin and Azeram	1 month	Intraepithelial clefts and acantholysis	Pemphigus vulgaris and vegetans
Tavares dos Santos et al., 2017 [22]	Clinical Case	1	------	Solitary lesions, blackberry lesions and erosions	Gum	Azathioprine and mesazalin	---------	Pseudoepitheliomatous hyperplasia with microabscesses + Granulomatous tissue with lymphocytic infiltrate	Fungal infection and syphilis
Ashley Eckel et al., 2017 [23]	Clinical Case	1	15 y	Pain, gingival bleeding, swelling and ulcers	Palate, cheeks and retromolar area	Corticosteroids	1 month	Discrete non-caseating granulomas, sialodenitis and plasmacytosis	Infectious diseases, drug reactions, nutritional
Anu Haarano et al., 2018 [24]	Transversal study	46	-------	Amygular cheilitis, ulcers, erythema, swelling	Mucosa, gingiva, lips, palate and floor of the mouth	Anti-TNF therapy, Azathioprine, 5-aminosalicylic acid and methotrexate.	----------	-------------------------	Orofacial granulomatosis
Saverio Capodiferro et al., 2019 [25]	Clinical Case	1	12 y	Gingivitis, fissures and angular cheilitis	Gums, lips and mucosa	Anti-inflammatories and immunosuppressants	-----------	Non-caseating granulomas	----------------
Miray Karakoyun et al., 2019 [26]	Clinical Case	1	16 y	Inflammation and ulcers	Lip and mucosa.	Methylprednisolone and mesalazine Azathioprine	3 months	--------------------------	-----------------
Mei Li Huang et al., 2020 [27]	Clinical case	1	11 y	Sores, swelling, cracking and tooth mobility	Oral mucosa and lips	Metronidazole, Infliximab Mercaptopurine	-----------	---------------------------	-----------------
Mohammad S. Alrasdan et al., 2021 [28]	Clinical Case	1	23 y	Canker sores	Buccal mucosa, uvula, pillars of the fauces and oropharynx	Adalimumab Prednisolone Azathioprine	3 months	Diffuse granulomatous inflammation	----------------
Sol de Boyang et al., 2021 [29]	Cohort study	18	-------	Caries, Periodontitis and bleeding	Teeth and periodontium	--------------------	------------	-------------------------	---------------
Franncesca Giaccaglia et al., 2021 [30]	Clinical Case	1	15 y	Erythema, inflammation and angular cheilitis	Gums and lips	Adalimumab Infliximad	------------	Inflammatory infiltration of submucosal lymphocytes and monocytes+ granulomatous aggregates	Sarcoidosis Granulomatosis orofacial
Colin E. McCorkle et al., 2021 [31]	Clinical Case	1	27 y	Gingival irritation and swelling of the lip	Lips and chin	Adalimumab Prednisona	4 months	Non-caseating submucosal granulomatous inflammation + lymphoid infiltrate	Sarcoidosis, Vasculitis, Neoplasms

**Table 3:** Results of the analysis of observational studies.

	Vavricka et al., 2013 [13]	Haarano et al., 2018 [24]	Boyang et al., 2021 [29]	Gale et al., 2014 [18]	Szczeklik et al., 2012 [11]
1	✔	✔	✔	✔	✔
2	✘	✔	✔	✘	✘
3	✘	✘	✘	✘	✘
4	✘	✘	✘	✘	✘
5	✔	✔	✔	✔	✔
6	✔	✔	✘	✔	✘
7	✔	✔	✔	✔	✔
8	✔	✘	✘	✘	✘
9	✔	✔	✔	✔	✘
10	✔	✔	✘	✔	✘
11	✔	✘	✘	✘	✔
Final puntuation	8	7	5	6	4
Risk of bias	moderate	moderate	high	moderate	high

**Table 4:** Results of the quality analysis of the clinical cases.

	McCorkle et al., 2021 [31]	Desmond et al., 2016 [20]	HM Preidl et al., 2014 [16]	Ciacci et al., 2014 [15]	Eckel et al., 2017 [23]	L. Woo 2015 [10]	Bruscino et al., 2012 [12]	Antunes et al., 2015 [19]	Capodiferro et al., 2019 [25]	Dos Santos et al., 2017 [22]	Atarbashi-moghada 2016 [21]	Karakoyun et al., 2019 [26]	Salek et al., 2013 [14]	Huang et al., 2020 [27]	Padmavathi et al., 2014 [17]	Giaccaglia et al., 2021 [30]	Alrasdan et al., 2021 [28]	Dos Santos et al., 2017 [22]	Atarbashi-moghadam 2016 [21]
1	✘	✔	✔	✔	✘	✔	✘	✘	✘	✘	✘	✔	✘	✘	✘	✔	✔	✘	✘
2	✔	✘	✔	✔	✘	✘	✘	✘	✔	✔	✔	✔	✔	✔	✘	✔	✔	✔	✔
3a	✔	✔	✔	✔	✔	✔	✘	✘	✔	✔	✔	✔	✔	✔	✔	✔	✔	✔	✔
3b	✔	✔	✔	✔	✔	✔	✘	✘	✔	✔	✔	✔	✔	✔	✔	✘	✘	✔	✔
3c	✘	✘	✔	✔	✔	✘	✘	✘	✔	✔	✔	✔	✔	✔	✔	✔	✘	✔	✔
3d	✔	✔	✔	✔	✔	✔	✘	✘	✔	✔	✔	✔	✔	✔	✔	✔	✔	✔	✔
4	✔	✘	✔	✔	✔	✔	✘	✘	✘	✔	✘	✔	✔	✔	✔	✔	✔	✔	✘
5a	✔	✔	✔	✔	✔	✔	✔	✔	✔	✔	✔	✔	✔	✔	✔	✔	✔	✔	✔
5b	✔	✔	✔	✔	✔	✔	✔	✘	✔	✔	✔	✔	✔	✔	✔	✔	✔	✔	✔
5c	✔	✔	✔	✔	✔	✔	✘	✔	✘	✔	✔	✔	✔	✔	✘	✔	✔	✔	✔
5d	✔	✘	✔	✘	✘	✔	✘	✘	✘	✔	✔	✘	✔	✔	✘	✔	✔	✔	✔
6	✔	✔	✔	✔	✔	✔	✔	✔	✔	✔	✔	✔	✔	✔	✔	✔	✔	✔	✔
7	✔	✔	✔	✔	✔	✔	✔	✔	✔	✔	✔	✔	✔	✔	✔	✔	✔	✔	✔
8a	✔	✔	✔	✔	✔	✔	✔	✔	✔	✔	✔	✔	✔	✔	✔	✔	✔	✔	✔
8b	✘	✘	✘	✘	✘	✘	✘	✘	✘	✘	✘	✘	✘	✘	✘	✘	✘	✘	✘
8c	✔	✘	✘	✘	✔	✔	✘	✔	✘	✘	✔	✘	✔	✔	✔	✔	✔	✘	✔
8d	✘	✘	✘	✘	✘	✘	✘	✘	✘	✘	✘	✔	✘	✘	✘	✘	✘	✘	✘
9a	✔	✔	✔	✔	✔	✔	✔	✔	✔	✔	✔	✔	✔	✔	✘	✔	✔	✔	✔
9b	✘	✘	✔	✔	✘	✘	✘	✔	✘	✔	✘	✔	✔	✘	✘	✘	✔	✔	✘
9c	✘	✘	✘	✘	✘	✘	✘	✘	✘	✘	✘	✘	✘	✘	✘	✘	✔	✘	✘
10a	✔	✔	✔	✔	✔	✔	✔	✔	✔	✔	✔	✔	✔	✔	✔	✔	✔	✔	✔
10b	✔	✔	✔	✔	✔	✔	✘	✘	✘	✔	✔	✔	✔	✔	✘	✘	✔	✔	✔
10c	✔	✘	✔	✔	✘	✘	✘	✘	✘	✘	✘	✘	✘	✘	✘	✘	✘	✘	✘
10d	✘	✘	✔	✘	✘	✘	✘	✘	✘	✘	✘	✘	✘	✘	✘	✘	✘	✘	✘
11a	✔	✔	✔	✔	✔	✔	✘	✔	✔	✔	✔	✔	✔	✔	✔	✔	✔	✔	✔
11b	✔	✔	✔	✔	✔	✔	✘	✘	✔	✔	✔	✔	✔	✔	✔	✔	✔	✔	✔
11c	✘	✘	✘	✔	✘	✘	✘	✔	✘	✔	✔	✔	✔	✔	✔	✔	✔	✔	✔
11d	✔	✔	✔	✔	✔	✔	✘	✘	✔	✔	✔	✔	✔	✔	✔	✔	✔	✔	✔
12		✘	✔	✔	✘	✔	✘	✘	✘	✘	✘	✘	✘	✘	✘	✘	✘	✘	✘
13	✔	✘	✔	✔	✘	✘	✘	✔	✘	✘	✘	✔	✘	✔	✘	✘	✘	✘	✘
Total	22	16	25	24	17	20	7	12	15	21	20	23	22	22	16	20	22	21	20
%	73.33%	53.33%	83.33%	80%	56.66%	66.66%	23.33%	40%	50%	70%	66.66%	76.66%	73.33%	73.33%	53.33%	66.66%	73.33%	70%	66.66%
